# Future self-imagery of young people in Sweden during the COVID-19 pandemic: an exploratory mixed methods analysis

**DOI:** 10.1007/s12144-022-04100-z

**Published:** 2022-12-20

**Authors:** Laura Singh, Clare J Rathbone, Michelle L. Moulds, Emily A. Holmes

**Affiliations:** 1 Department of Psychology, Uppsala University, Box 1225, 751 42 Uppsala, Sweden; 2 Swedish Collegium for Advanced Study, Uppsala, Sweden; 3 Centre for Psychological Research, Oxford Brookes University, Oxford, UK; 4 School of Psychology, The University of New South Wales, UNSW Sydney, Kensington, Australia

**Keywords:** COVID-19, Future imagery, Stressful event, Trauma, Optimism, Mental imagery

## Abstract

Global restrictions imposed during the COVID-19 pandemic significantly limited the capacity to plan for the future. Little is known about young people’s future self-images and the impact the COVID-19 pandemic may have had upon them. Given evidence that the ability to imagine a positive future can be protective for mental health, research into the impact of the pandemic on future self-imagery is needed. In two studies, we therefore explored the consequences of the COVID-19 pandemic for future self-imagery in an unselected sample of young people in Sweden, specifically: (a) how content and characteristics of future self-images changed from before to during the pandemic, and (b) how such change was related to trauma history, intrusive memories of COVID-19 media footage, past time perspective and optimism (assessed with questionnaires/intrusive memory diary). Future self-images before and during the pandemic were assessed using the ‘I Will Be’ task (*N*_Study1_=74; *N*_Study2_=99). A mixed methods design, combining quantitative analysis, qualitative content coding and thematic analysis was used. Exploratory results of Study 1 indicated that future images were rated as less positive during than before the pandemic and that this reduction was less pronounced in people with higher optimism. Results were replicated in an independent sample (Study 2, collected later during the pandemic). In conclusion, whilst the findings are preliminary and emerged from an unselected sample, they prompt the suggestion that brief, novel interventions which aim to bolster positive future imagery may hold promise as a scalable means by which to enhance mental health for young people in the context of the COVID-19 pandemic.

**Supplementary information:**

The online version contains supplementary material available at 10.1007/s12144-022-04100-z.

## Introduction

The ability to imagine the future is a fundamental skill. Images of positive events in the future inspire hope, motivate goal-setting, and guide us in taking steps to achieve them (Heise et al., 2022; Kress & Aue, 2017). The global restrictions imposed to mitigate the spread of COVID-19 significantly limited the capacity to plan for the future. The possibility that disruptions to future planning influenced individuals’ self-imagery has, to the best of our knowledge, not been studied to date. Adverse mental health consequences of the pandemic have been documented across the population (Holmes et al., 2020; McCracken et al., 2020), including in young people (Creswell et al., 2021; Racine et al., 2021). Whilst recent research has examined the association between how people remember pandemic-related events and *thought* about the future and psychological well-being (Niziurski & Schaper, 2021), such work has focused on future thinking (i.e., verbal thoughts) rather than future imagery. Given evidence that the ability to *imagine* a positive future can be protective for mental health, research into the impact of the pandemic on future self-*imagery* is needed (Holman et al., 2022; Holman & Grisham, 2020).

Links between psychological wellbeing and future imagery are well-established. For example, depression symptoms are associated with a deficit in generating vivid images of positive future events (Holmes et al., 2008) and a lack of spontaneous positive future imagery is linked to diminished mood, motivation and optimism (Ji et al., 2021a, b). Imagery of the future can also be disrupted following traumatic or stressful events. For instance, focusing on the past rather than the future is related to distress following a traumatic event (Holman & Silver, 1998). Further, Kleim et al. (2014) found that individuals with posttraumatic stress disorder (PTSD) provided fewer specific images of future events in response to positive (but not negative) cues relative to trauma-exposed individuals without PTSD. These findings converge on the suggestion that future imagery is compromised in traumatised individuals.

Recurrent and distressing intrusive memories of a traumatic event are a key symptom of PTSD (American Psychiatric Association, 2013). The constructive episodic simulation hypothesis (Schacter & Addis, 2007) holds that recalling the past and imagining the future recruit overlapping cognitive and neural processes (Conway et al., 2019). It follows that if a traumatised individual is ‘stuck in the past’ such that they experience recurrent intrusive memories of a trauma, their capacity to vividly imagine the future will be restricted.

Several questions await empirical test. To our knowledge, whilst some research has examined future imagery in PTSD, no study has examined *self*-related future imagery in the context of an ongoing trauma or stressor. Evidence that individuals with PTSD perceive their trauma as being at the centre of their identity (Brown et al., 2010) and view their past (pre-trauma) self more favourably than their current or future selves (Brown et al., 2011) raises the possibility of an association between psychological trauma and future self-imagery. Further, no research has investigated future self-imagery soon after a trauma, whether individuals’ future self-imagery changes over the course of experiencing a stressor such as the COVID-19 pandemic, nor whether such changes are associated with trauma-related sequelae (e.g., intrusive memories) or optimism.

To fill these gaps in the literature, we used a naturally occurring (rather than laboratory) stressor, the COVID-19 pandemic, to explore the consequences of ongoing trauma for future self-imagery in young people. We used a novel task assessing future *imagery* rather than future *thinking* and conducted two studies during different stages of the COVID-19 pandemic. Data for study 1 was collected during the second and third wave of the pandemic in Sweden and used to explore the effect of the pandemic on future self-imagery, as well as the relationship between changes in self-imagery and other (trauma-related) variables. Data for Study 2 was collected during the fourth wave of the COVID-19 pandemic in Sweden and used as a test of replication (for the hypotheses generated in Study 1 and pre-registered prior to data analysis) on an independent dataset.

### Exploratory research questions

How do the content and characteristics of young people’s future self-images change from before to during the COVID-19 pandemic in Sweden? How is such change related to other (trauma-related) variables (i.e., trauma history, intrusive memories of COVID-19 media footage, past time perspective and optimism)?

## Study 1 methods

Data were collected in the context of a randomized two-group experimental study investigating the feasibility of delivering the trauma film paradigm, a brief cognitive intervention/control task, and a daily intrusion diary remotely/digitally during the COVID-19 pandemic (ClinicalTrials.gov ID: NCT04608097, prospectively registered on 29/10/2020) (Annersand, 2021). Experimental protocols were based on previous studies in our laboratory, and adapted for remote delivery with researcher guidance to adhere to guidelines on social distancing during the COVID-19 pandemic. Study procedures were completed electronically using the data collection tool Qualtrics (Qualtrics, 2015). We employed a mixed methods design, combining quantitative analysis, qualitative content coding and thematic analysis.

### Participants

An unselected sample of seventy-four participants (50 women, 24 men; 18 to 45 years old, *M* = 23.84, *SD* = 4.75) was recruited via social media advertisements targeting Swedish university students between October 2020 and March 2021 (i.e., during the second and third wave of the COVID-19 pandemic in Sweden). Inclusion criteria were: aged 18–65; fluent in spoken and written Swedish; willing to watch a video containing emotional, distressing footage; having access to an internet enabled smartphone/computer. Exclusion criteria were: having participated in a study in which similar stimuli were used; currently receiving treatment for a mental health problem (e.g., depression, anxiety, ADHD, addiction), including psychological therapy, counselling or medication; neurological illness (e.g., epilepsy); planning to undertake a stress-inducing examination (e.g., university examination or driving test) during the week of study participation.

All participants provided their digital written and informed consent prior to participating and were informed that they could end their participation at any time. All procedures were approved by the Swedish Ethical Review Authority (approval number: 2020–03991) and carried out in accordance with the ethical guidelines of the Declaration of Helsinki (World Medical Association, 2018). Participants were reimbursed with a book voucher (value 200 Swedish crowns).

### Measures

#### Modified I will be task

A modified version of the I Will Be Task (Chessell et al., 2013; Rathbone et al., 2011) was used to measure future self-imagery before and during the COVID-19 pandemic. Participants were instructed to generate three ‘I will be’ statements (e.g., I will be *more active*) describing how they imagined themselves in the future (future self-identity) before the COVID-19 pandemic in relation to social/occupational/other important situations (free text response). Participants were then instructed to indicate which of these three future identities had been most affected by the COVID-19 pandemic (if none had been affected by the pandemic, they were instructed to choose their most important future identity). Next, participants were asked to briefly describe a specific mental image they had of themselves in the future in relation to this identity *before the COVID-19 pandemic.* While keeping this image in mind, participants rated its vividness (Likert scale from 1 ‘not vivid at all’ to 10 ‘very vivid’), valence (1 ‘very negative’ to 10 ‘very positive’) and perspective (‘through own eyes’ or ‘as if seeing oneself from the outside’), and indicated the presence of other people in the image (‘none’ or ‘__ other people were present’), the regularity of rehearsal of the image (1 ‘never’ to 10 ‘very regularly’), the likelihood that it would become reality (1 ‘not at all likely’ to 10 ‘very likely’), and how old they will be in the image. Next, participants briefly described how the COVID-19 pandemic had impacted the image. Last, participants were asked to briefly describe a specific mental image they had of themselves in relation to the same identity *now, during the COVID-19 pandemic* and complete the same ratings while keeping this second image in mind.

#### Life Event Checklist for DSM-5(LEC-5) (Weathers et al., 2013)

Trauma history was measured with the LEC-5, a 17-item self-report questionnaire used to screen for potentially traumatic events over the lifetime. For each item/event respondents indicate one of the following: ‘happened to me’, ‘witnessed it’, ‘learned about it’, ‘part of my job’, ‘not sure’ or ‘doesn’t apply’. A total score was calculated by adding the number of events endorsed as ‘happened to me’, ‘witnessed it’, or ‘part of my job’ (see Lebeaut et al., 2020; Singh et al., 2021). Higher scores indicate a higher number of prior traumatic events. Reliability in the current sample was α = 0.728.

#### Digital intrusive memory diary (Singh et al., 2021)

The number of intrusive memories related to the trauma film was assessed in a seven-day digital intrusive memory diary (adapted from a previously used pen-and-paper version) (James et al., 2015; Lau-Zhu et al., 2019). Once daily participants received an email with a link and were asked to indicate how many intrusive memories they experienced since the last registration using a drop-down menu (0 to 30) and to provide a brief description of each memory. Intrusive memories were defined as mental images (including other senses, e.g., sounds) related to the trauma film that come to mind spontaneously and are unwanted, and typically short, fragmented or fleeting. Participants were told not to record memories that were retrieved deliberately or that were purely verbal. Participants were given written and verbal instructions (via video) about how to complete the diary; their understanding of the instructions, the definition of intrusive memories and how to complete the diary were checked by a researcher in a follow-up phone call. Intrusion diary data has been shown to correspond with scores on the Intrusion subscale of the Impact of Event Scale (Horowitz et al., 1979)/Impact of Event Scale – Revised (Weiss et al., 1997) (moderate correlation, r = .487), a widely used questionnaire measure of intrusion symptoms (Singh et al., 2022).

#### Time Perspective Questionnaire (TPQ) (Holman et al., 2016)

Time perspective was measured with the Time Perspective Questionnaire, an 8-item self-report questionnaire assessing the extent to which respondents agree with statements about the past (e.g., ‘It is hard for me to forget unpleasant images of my past’), present (e.g., ‘I try to live one day at a time’) and future (e.g., ‘My plans about the future are pretty well laid out’) on a 5-point scale from 1 ‘not at all true’ to 5 ‘completely true’. Three subscale scores were calculated by summing item scores for past, present and future perspective. Higher scores indicate higher levels of past, present, and future perspective. Reliability was α = 0.620 for future time perspective, α = 0.808 for present time perspective, and α = 0.773 for past time perspective.

#### Future Expectancy Scale (FES) (Peters et al., 2016)

Optimism was measured using the future expectancy scale, a self-report questionnaire assessing expectancies about 10 positive future life events (e.g., ‘You will live a long life’). Each event is rated regarding how likely it is to happen in one’s future on a 7-point scale ranging from 1 (‘not at all likely’) to 7 (‘extremely likely’). A total score is calculated by summing all items. Higher scores indicate more optimistic/positive future expectancies. Reliability in the current sample was α = 0.881.

### Procedure

After providing written and informed consent, participants completed baseline questionnaires (e.g., demographics, LEC-5). Participants then viewed a trauma film consisting of 12 short film clips depicting serious illness and death in relation to the COVID-19 pandemic (e.g., seriously ill or dead patients in a hospital, desperate hospital staff, relatives mourning their family members who died from COVID-19; all clips were derived from the public domain such as news/media footage and compiled by research assistants in our lab). Next, participants completed the intervention or control task investigated within the wider project (not relevant for the current study) and received instructions as to complete the digital intrusive memory diary. At the follow-up session one week later, participants completed the I Will Be Task (main outcome of the current study), as well as additional questionnaires (e.g., TPQ, FES; see flow chart in Supplementary Fig. 1).

### Data analysis

The main outcome of the current study was the I Will Be task, which was administered at the end of all study procedures and unrelated to the group design of the wider project. Task instructions were anchored to effects of the COVID-19 pandemic in general, rather than to any aspect of the other study procedures (e.g., experimental manipulations – trauma film, intervention/control task). Accordingly, the whole sample was included in the current ancillary analysis. All analyses were performed using JASP for Windows, version 0.14.1 (JASP Team, 2021). Coding and thematic analyses were conducted in Microsoft Excel for Windows (2013).

#### Qualitative analyses

Qualitative data (from the I Will Be Task) was analysed using different coding schemes (see Supplementary Tables S1-S3). All coding was conducted independently by two research assistants. Any disagreements between the coders were discussed with an additional senior researcher (LS), and decisions about final category allocation were made jointly.

A coding scheme developed by Rathbone et al. (2016) was used to group participants’ ‘I Will Be’ (future identity) statements related to social, occupational and other important situations into one of 28 possible categories (e.g., ‘marriage’, ‘parenthood’). Coders were instructed to only code the first statement if more than one category was described within an ‘I Will Be’ statement (e.g., ‘I will be a husband and father’). Cohen’s kappa indicated substantial to almost perfect agreement (*κ* = 0.66 – 0.85) between the independent coders (see Supplemental Table S4 for Kappa values for each part of the coding schemes).

To analyse whether and how the content of participants’ future images related to their ‘I Will Be’ statement changed from *before* to *during* the pandemic, descriptions were coded into three categories: (1) no change (the image during the pandemic is the same as before the pandemic); (2) the image content has changed to a different/adaptive way forward (e.g., the image has been adjusted to one that is possible under pandemic conditions but is similar in valence/quality), (3) the image content is weaker (e.g., the image is described as less clear, less likely to happen, to happen further away in the future) or less positive than before the pandemic. These three categories were established by LS and CR using a bottom-up approach of identifying key topics in the raw data. Cohen’s kappa indicated substantial agreement (κ = 0.69) between the independent coders.

Furthermore, descriptions of participants’ future images were coded for specificity using a coding framework developed by LS and CR for the current study (loosely based on Baddeley & Wilson, 1986). Specifically, descriptions were coded for the presence or absence of the following five features: (1) Is a *specific time constrained image/event* described? (2) Does the description include information on *who* is in the image? (3) Does the description include information on *where* the image/event takes place? (4) Does the description include information on *when* the image/event takes place? (5) Does the description include information on *what* is happening in the image/event? Both coders were blind to whether the image description referred to *before the COVID-19 pandemic* or *during the pandemic* and the dataset used for coding did not contain this information, i.e., statements were listed alphabetically, not organised by time (although note that the content of some image descriptions inevitably revealed whether it referred to before or during the pandemic). Cohen’s kappa indicated slight to almost perfect agreement (κ = 0.19 – 0.85) between the independent coders.

#### Quantitative analyses

Descriptive analyses were conducted to present frequencies of different categories of future self-identities that had changed most during the COVID-19 pandemic and whether/how the content of mental images related to those future identities had changed. Characteristics of the mental images rated in relation to how they were experienced before and during the pandemic were compared using non-parametric Wilcoxon Signed-Rank Tests (given that data were not normally distributed). Effect sizes are reported as Rank-Biserial Correlations. Associations between changes in characteristics of mental images and other (trauma-related) variables were explored using Spearman’s Rho Correlations. Differences in other trauma-related variables between participants whose future image content had been categorized as no change, different/adaptive way forward, and weaker/less positive were explored using one-way ANOVAs (if data were normally distributed) or non-parametric Kruskal-Wallis Tests. Post-hoc pairwise comparisons were done using Tukey correction (for one-way ANOVAs) and Bonferroni correction (for Kruskal-Wallis Tests) for multiple tests. A two-tailed alpha level of 0.05 was used for all analyses. Given the exploratory nature of the study, results are interpreted with caution. Effect sizes are reported as Cohen’s d (t-tests) and partial η2 (one-way ANOVAs).

#### Thematic analysis

Relevant themes in participants’ open responses to the question ‘How did the COVID-19 pandemic impact your future image?’ were explored using thematic step-wise analysis (based on Braun & Clarke, 2006) conducted by two research assistants and supervised by a senior researcher (LS). The analysis (guided by the research question) included the following 5 steps: (1) Familiarization with the data, (2) creating codes from the raw data (i.e., highlighting relevant sections of text using the same colour for similar aspects and identifying matching codes), (3) grouping codes into themes (i.e., identifying overlapping patterns among codes and generating broader themes), (4) reviewing and revising themes by going back to the raw data, (5) defining and naming themes.

## Study 1 results

### Most impacted future identities among young people during the COVID-19 pandemic

When asked which future identity has been most impacted by the COVID-19 pandemic, most participants (*n* = 32, 43.2%) chose their future identity in ‘other important situations’, 23 participants (31.1%) chose their future identity in ‘social situations’, and 19 participants (25.7%) chose their future identity in ‘occupational situations’. Table 1 presents specific categories and examples of future identities participants described as having been most impacted by the pandemic.

**Table 1:** Content of future self-identities that had been impacted most by the COVID-19 pandemic in study 1

Category	Count (*n*)	%	Examples described by participants (*I Will Be…*)
Job – specific	11	14.9	*… a good doctor*
Self-improvement	9	12.2	*… more socially active*
Activities	7	9.5	*… a triathlete*
Job – general	7	9.5	*… good at my job*
Friendship	6	8.1	*… a person with many friends*
Health	6	8.1	*… more physically fit*
Happy	5	6.8	*… a happy person*
Marriage	5	6.8	*… married to a pretty woman one day*
Parenthood	5	6.8	*… a mum (hopefully a good one)*
Other	3	4.1	*… part of a bigger social context*
Move	2	2.7	*… ending my long-distance relation and live together with my boyfriend*
Fall in love	1	1.4	*… loved*
Family	1	1.4	*… a good aunt*
Grandchildren	1	1.4	*… a grandfather*
Learn language	1	1.4	*… fluent in French, travelling, knowing what I want to do with my life later on*
Relationship	1	1.4	*… a good boyfriend who is going out and traveling with my girlfriend*
Skill development	1	1.4	*… developing within my sport*
Successful	1	1.4	*… more successful*
Trait – general	1	1.4	*… the same*

Please see Supplementary Table S5 for specific categories and examples of future identities participants described in relation to ‘social situations’, ‘occupational situations’ and ‘other important situations’

### Changes in content of mental images related to those future identities during the pandemic (researcher coded)

30 participants (40.5%) described the same image content or stated that the image had not changed (e.g., ‘*No difference’*, ‘*It is the same as before. I’m surrounded by my family and we grow parts of our food at home*’). 23 participants (31.1%) described images that had changed to a different/adaptive (with regards to the pandemic) way forward (e.g., ‘*Arranges events in a creative way, e.g., outside/online*’, ‘*I see myself having dinners with my friends over zoom*’). 21 participants (28.4%) described images that were weaker/less positive (e.g., ‘*Unsure, no idea of when we can travel again or what the future looks like. Hard to get a clear picture*’, ‘*I still see myself as a doctor in the future, but it is more stressful. I look exhausted*’).

### Changes in characteristics of mental images related to future identities during the pandemic

Changes in specificity of the mental images participants described in relation to the future identity that had been most impacted by the pandemic (rated by the researcher) and other image characteristics (rated by the participants) are summarized in Table 2 below. Mental images were rated as less positive (large effect according to Cohen, 1988), more vivid, and with fewer other people present (medium effects) during as compared to before the pandemic.

**Table 2:** Characteristics of mental images related to future identities before and during the COVID-19 pandemic in study 1

	Before pandemic		During pandemic		Analysis		
Categorical variables	*n*	%	*n*	%			
*Perspective*							
Through own eyes	27	36.5	32	43.2			
As an observer	47	63.5	42	56.8			
Presence of other people in the image							
Yes	25	33.8	32	43.2			
No	49	66.2	42	56.8			
Continuous variables	Median (Range)	Mean (SD)	Median (Range)	Mean (SD)	W	*p*	Effect size
Number of other people present^a^	2.25^b^ (0–40)	4.32 (7.19)	1.00^c^ (0-100)	3.61 (12.21)	393.50*	0.043	0.403
Specificity^d^	3.00 (0–5)	2.73 (1.31)	3.00 (0–5)	2.64 (1.45)	701.00	0.719	0.057
Vividness^e^	7.00 (1–10)	6.35 (2.20)	7.00 (1–10)	6.89 (2.17)	525.00*	0.039	− 0.318
Valence^f^	9.00 (3–10)	8.81 (1.56)	8.00 (1–10)	7.35 (2.67)	1062.50***	< 0.001	0.603
Regularity of rehearsal^e^	7.00 (1–10)	6.42 (2.47)	6.00 (1–10)	5.85 (2.51)	1290.50	0.092	0.241
Likelihood of image becoming reality^e^	8.00 (1–10)	7.36 (2.40)	8.00 (2–10)	7.76 (1.97)	619.50	0.287	− 0.166
Distance to current age (in years)	4.00 (0–68)	6.34 (9.69)	3.00 (0–28)	5.53 (6.39)	422.50	0.922	− 0.019

^a^Some participants reported non-numerical answers that could not be included in the analysis (e.g., ‘*several’*, ‘*my family and friends’*). ^b^based on numerical answers obtained from *n* = 64. ^c^based on numerical answers obtained from *n* = 69. ^d^based on researcher coding (scale from 0 to 5) of image description. ^e^based on participant rating (scale from 1 ‘*not at all*’ to 10 ‘*extremely’*). ^f^based on participant rating (scale from 1 ‘*very negative*’ to 10 ‘*very positive’*). ***p < .001, **p < .01, *p < .05

### Association between change in valence ratings of mental images and other (trauma-related) variables

**Fig. 1:**
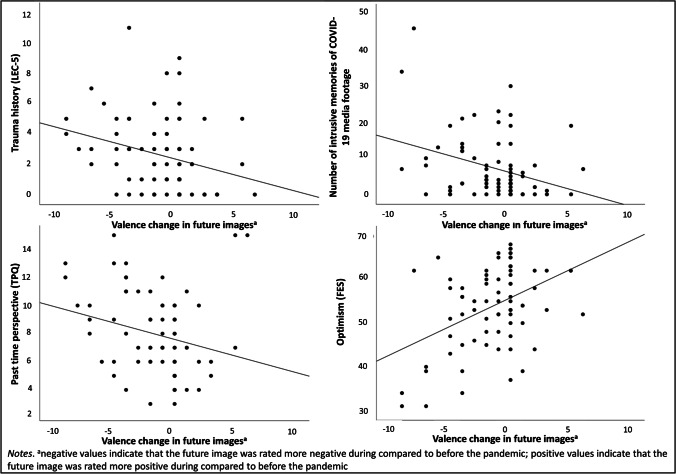
Association between valence change in future images from before to during the COVID-19 pandemic and trauma history (LEC-5), number of intrusive memories of COVID-19 media footage, past time perspective (TPQ), and optimism (FES) in study 1

The more prior trauma participants had experienced (LEC-5), the more negative their future self-image became from pre-pandemic to during the pandemic (*r* = − .317, *p* = .006). Similarly, the higher number of intrusive memories of COVID-19 media footage participants experienced (*r* = − .266, *p* = .022), and the higher ratings of past time perspective they reported (*r* = − .328, *p* = .004), the more negative their future self-image became from pre-pandemic to during the pandemic. In contrast, the more optimistic participants were, the less negative their future image became from pre-pandemic to during the pandemic (*r* = .327, *p* = .004); see Fig. 1 below.

### Change of mental image content and other (trauma-related) variables

When comparing participants whose future image content had not changed, changed to a different/adaptive way forward, or became weaker or less positive, there was a significant between-group difference for the number of prior traumas, (*H*(2) = 8.775, *p* = .012). Specifically, participants whose future image content had not changed during the pandemic reported significantly fewer prior traumas than participants whose future image content was weaker/less positive during compared to before the pandemic (*p* = .005). Similarly, there was a significant difference in past time perspective between categories (*H*(2) = 7.892, *p* = .019), such that participants whose future image content was weaker/less positive (*p* = .017) and those participants whose future image content changed to a different/adaptive way forward (*p* = .046) during compared to before the pandemic reported significantly higher past perspective than those whose future image content had not changed.

**Fig. 2:**
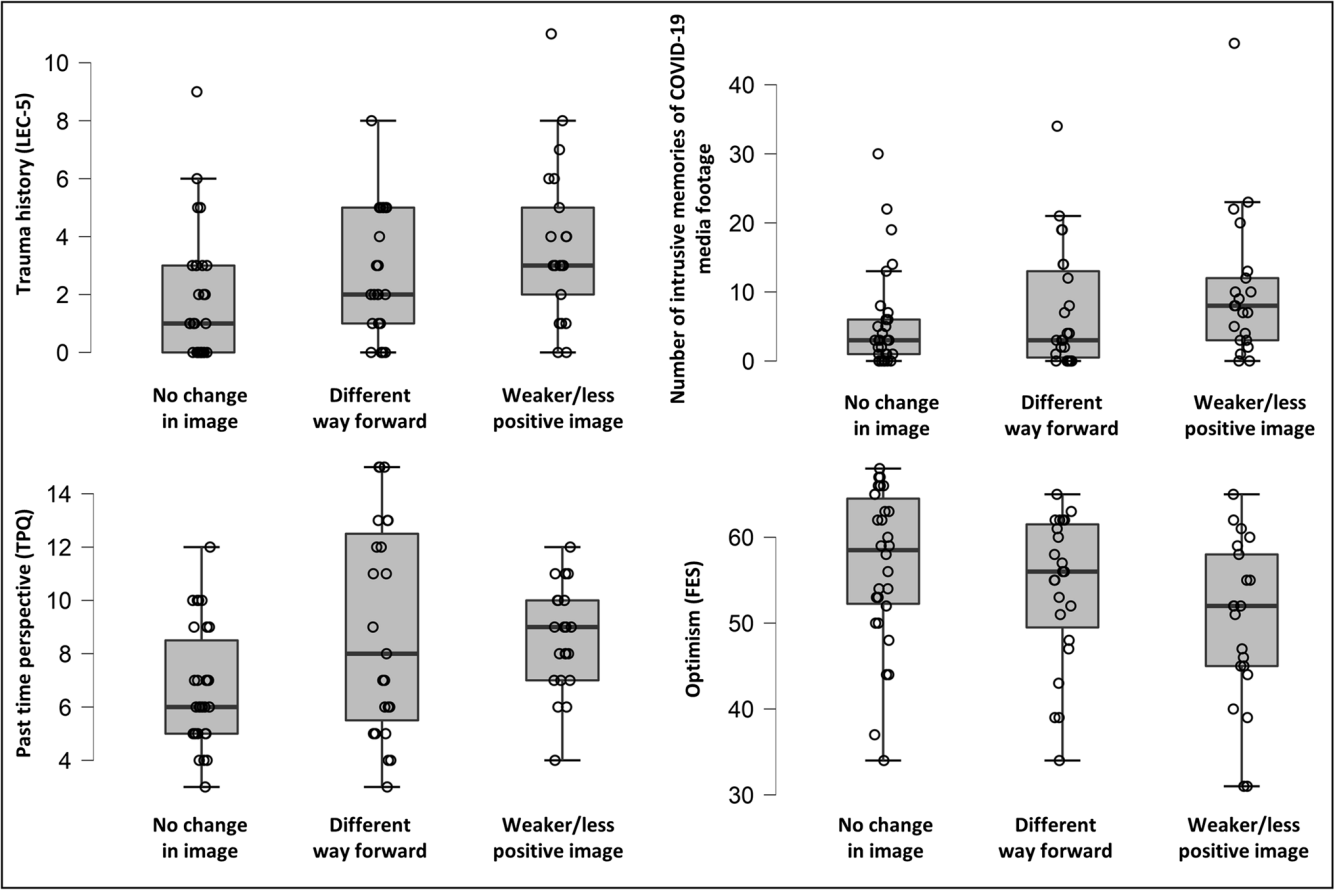
Boxplots illustrating change of mental image content (researcher-rated) and trauma history (LEC-5), number of intrusive memories of COVID-19 media footage, past time perspective (TPQ), and optimism (FES) in study 1

Furthermore, there was a significant difference in optimism between categories (*H*(2) = 6.643, *p* = .036). Specifically, participants whose future image content had not changed during the pandemic reported significantly higher optimism than participants whose future image content was weaker/less positive during compared to the pandemic (*p* = .015). No differences emerged for the number of intrusive memories of COVID-19 media footage (*H*(2) = 4.952, *p* = .084), see Fig. 2 for details.

### Themes in how the COVID-19 pandemic impacted young people’s future images

A thematic analysis conducted to identify themes in participants’ descriptions of how the pandemic has impacted the mental images related to their future identities revealed four main themes, each including several subthemes (see Table 3 for details and participant quotes).

**Table 3:** Themes identified in descriptions of how the COVID-19 pandemic impacted future images in study 1

Themes	Subthemes	Example quotes
The COVID-19 pandemic’s effect on the perception and cognitive aspects regarding future images	Vividness of the image	*It is harder to imagine*
	Time perspective	*It has been postponed*
	Rethinking/restructuring the future image	*I see the image differently*
	Replaced future image	*The future image has been completely changed*
	Unchanged future image	*I see it the same way*
The COVID-19 pandemic’s effect on emotions and attitudes regarding future images	Negative emotions	*Haven’t been less stressed since you worry about different things during the pandemic*
	Changed emotions	*I now see it as a stressful situation*
	Optimism	*I think it could still happen*
	Pessimism	*It will probably not come true*
Consequences of the COVID-19 pandemic on aspects of social life	Impact on relationships	*Difficult to date someone*
	Impact on friendships	*Drifted apart instead of getting closer to each other*
	Impact on social gatherings	*It will maybe forever be harder to meet in groups*
	Impact on work	*Easier to have a flexible job/easier to work from home*
	Impact on the way people socialize	*All education has been online*
Consequences of the COVID-19 pandemic on aspects of the society	A changed world	*Structural unemployment and recession* *Difficult to do things in other countries*
	Impact of COVID-19 related restrictions	*We had to cancel all practices due to the pandemic*

## Study 1 discussion

Study 1 aimed to explore whether the content and characteristics of young people’s future self-images changed from before to during the COVID-19 pandemic in Sweden, and how such change was related to other trauma-related variables (i.e., trauma history, intrusive memories of COVID-19 media footage, past time perspective) and optimism. Exploratory analyses indicated that the content of future self-images (researcher-rated) had not changed for c. 40% of participants, whereas it had changed to a different/adaptive way forward (c. 30%) or a weaker/less positive image (c. 30%) for the remaining participants. Participants rated their future self-images as less positive and more vivid and with fewer other people present during compared to before the COVID-19 pandemic. Greater changes in valence of future images (i.e., toward a more negative version) were associated with more prior trauma, more intrusive memories of COVID-19 media footage, and higher ratings of past time perspective. In contrast, the more optimistic participants were, the smaller the change in their ratings of the valence of future images (i.e., toward a more negative version). Similar patterns emerged between the researcher-rated categories regarding changes in participants’ future image content. Specifically, participants whose future images changed to a weaker/less positive version reported more prior trauma, more past time perspective and less optimism than participants whose future images did not change from before to during the pandemic. We tested whether these findings were replicated in an independent dataset in Study 2.

## Study 2 methods

Data were collected in the context of a second, independent randomized two-group experimental trial using similar procedures, with slight adaptations to the procedures of Study 1 (ClinicalTrials.gov ID: NCT05063825, prospectively registered on 01/10/2021) (Wallin, 2021). Unless otherwise specified, methods and materials used in Study 2 were identical to Study 1.

### Participants

Ninety-nine non-clinical participants (71 women, 25 men, 2 non-binary, 1 other gender identity; 18 to 65 years old, *M* = 25.73, *SD* = 7.57) recruited between October and December 2021 (i.e., during the fourth wave of the COVID-19 pandemic in Sweden) took part.

### Measures

#### Life Event Checklist for DSM-5 (LEC-5) (Weathers et al., 2013)

Reliability in the Study 2 sample was α = 0.829.

#### Time Perspective Questionnaire (TPQ) (Holman et al., 2016)

Reliability was α = 0.674 for future time perspective, α = 0.778 for present time perspective, and α = 0.852 for past time perspective.

#### Future Expectancy Scale (FES) (Peters et al., 2016)

Reliability in the Study 2 sample was α = 0.901.

### Procedure

Most study procedures were identical to Study 1. However, in Study 2, participants who rated their distress related to the COVID-19 trauma film as less than 5 (on a scale from 0 to 10) were excluded from the study after film viewing.

### Data analysis

#### Qualitative analyses

Coding of changes in the content of future images was conducted independently by a research assistant and LS. Any disagreements between coders were discussed and decisions about final category allocation were made jointly. Cohen’s kappa indicated moderate agreement (κ = 0.45) between the independent coders.

#### Quantitative analyses

Based on the results of Study 1, we pre-specified the analyses we planned to conduct on the independent dataset of Study 2 (see https://osf.io/pvx87/ for the statistical analysis plan published prior to Study 2 data analysis on 09-02-2022).

## Study 2 results

### Changes in content of mental images related to those future identities during the pandemic (researcher coded)

48 participants (48.5%) described the same image content or stated that the image had not changed (e.g., ‘*I have the same image as before, I will be a person with many interests’*, ‘*The image looks the same*’). 35 participants (35.4%) described images that had changed to a different/adaptive (with regards to the pandemic) way forward (e.g., ‘*I will sit in a zoom meeting with people from other countries*’, ‘*I see myself having few but very close friends*’). 16 participants (16.2%) described images that were weaker/less positive (e.g., ‘*Very unclear, but I tend to seeing myself lonely without belonging to a group*’, ‘*Sitting home alone and not being able to meet so many people because of the restrictions*’).

### Changes in characteristics of mental images related to future identities during the pandemic

Changes in image characteristics (rated by the participants) are summarized in Table 2. Mental images were rated as less positive, more vivid, with fewer other people present, and with oneself being older compared to one’s current age (medium effects) during as compared to before the pandemic (Table 4).

**Table 4:** Characteristics of mental images related to future identities before and during the COVID-19 pandemic in Study 2

	Before pandemic		During pandemic		Analysis		
Categorical variables	*n*	%	*n*	%			
Perspective							
Through own eyes	30	30.3	55	55.6			
As an observer	69	69.7	44	44.4			
Presence of other people in the image							
Yes	66	66.7	62	62.6			
No	33	33.3	37	37.4			
Continuous variables	Median (Range)	Mean (SD)	Median (Range)	Mean (SD)	W	*p*	Effect size
Number of other people present^a^	1.00^b^ (0-60000)	679.36 (6359.45)	1.00^c^ (0-100)	3.51 (11.10)	717.00*	0.010	0.448
Vividness^d^	7.00 (1–10)	6.44 (2.21)	7.00 (1–10)	6.90 (1.98)	994.50*	0.021	− 0.302
Valence^e^	9.00 (5–10)	8.58 (1.46)	8.00 (1–10)	7.65 (2.34)	1785.50**	0.001	0.437
Regularity of rehearsal^d^	7.00 (2–10)	6.30 (2.19)	6.00 (1–10)	5.91 (2.39)	1492.50	0.086	0.236
Likelihood of image becoming reality^d^	8.00 (1–10)	7.39 (2.35)	7.00 (1–10)	7.23 (2.07)	1450.50	0.442	0.104
Distance to current age (in years)	1.00 (-3-40)^f^	3.43 (6.71)	2.00 (-1-24)^g^	3.75 (4.11)	694.00***	< 0.001	− 0.457

^a^Some participants reported non-numerical answers that could not be included in the analysis (e.g., ‘*several’*, ‘*husband and family’*). ^b^based on numerical answers obtained from *n* = 89. ^c^based on numerical answers obtained from *n* = 88. ^d^based on participant rating (scale from 1 ‘*not at all*’ to 10 ‘*extremely’*). ^e^based on participant rating (scale from 1 ‘*very negative*’ to 10 ‘*very positive’*). ^f^Given that during data collection of Study 2 the pandemic had been going on for several years, participants’ age in the future images they imagine before the pandemic could be younger than their current age, thus leading to negative values for distance to current age (*n* = 13). ^g^Two participants indicated that they are one year younger in their future image than they currently are, thus indicating that they misunderstood the task and imagined a past image. Excluding these participants from the analysis did not change the result, which is why we opted to include all data. ***p < .001, **p < .01, *p < .05

### Association between change in valence ratings of mental images and other (trauma-related) variables

**Fig. 3:**
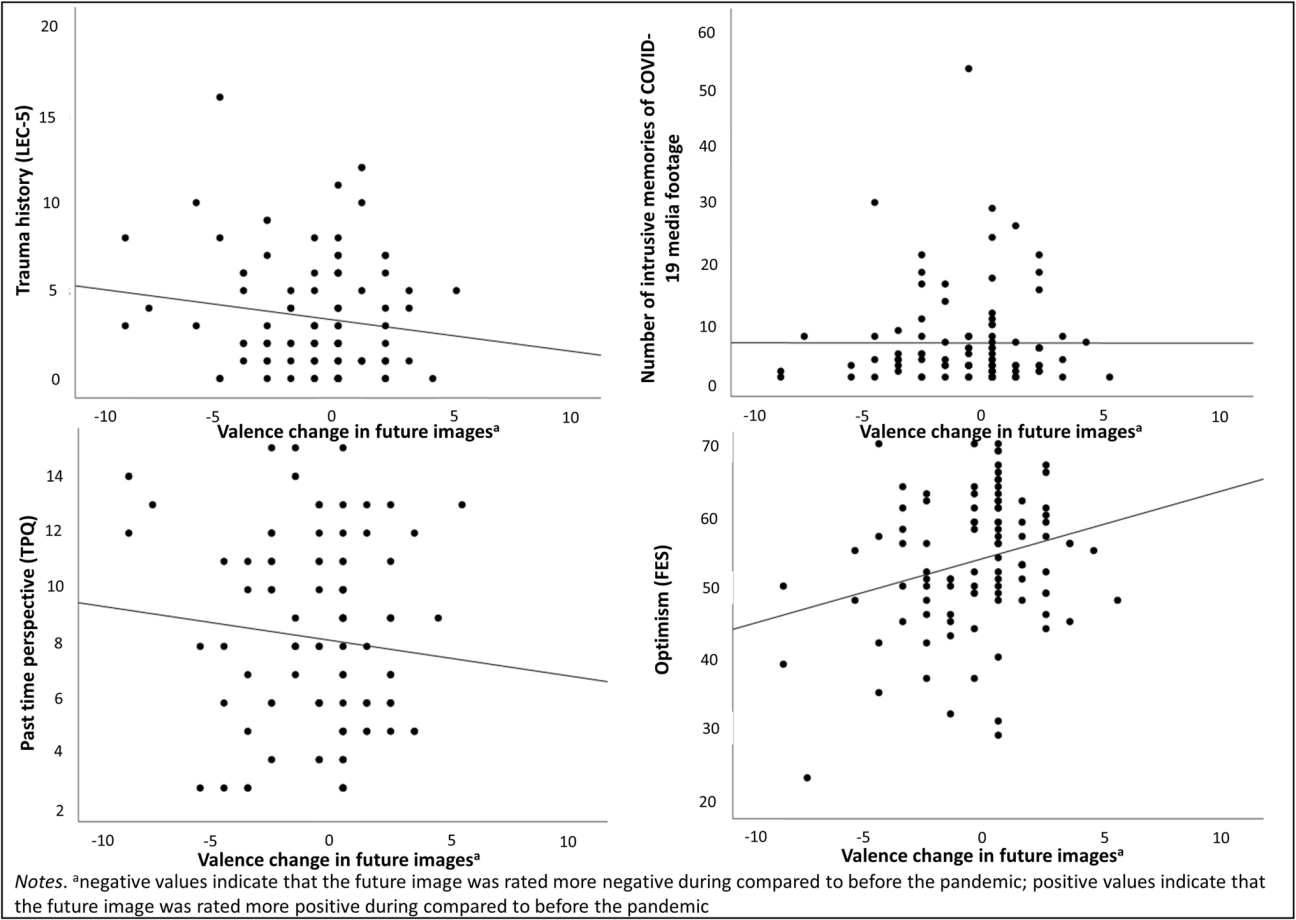
Association between valence change in future images from before to during the COVID-19 pandemic and trauma history (LEC-5), number of intrusive memories of COVID-19 media footage, past time perspective (TPQ), and optimism (FES) in study 2

No significant associations emerged between the change in participants’ valence rating from before to during the pandemic and their trauma history (*r* = − .094, *p* = .354), number of intrusive memories of COVID-19 media footage (*r* = − .041, *p* = .688) or ratings of past time perspective (*r* = − .087, *p* = .394). However, the more optimistic participants were, the less negative their future image became from pre-pandemic to during the pandemic (*r* = .203, *p* = .044); see Fig. 3 below.

### Change of mental image content and other (trauma-related) variables

**Fig. 4:**
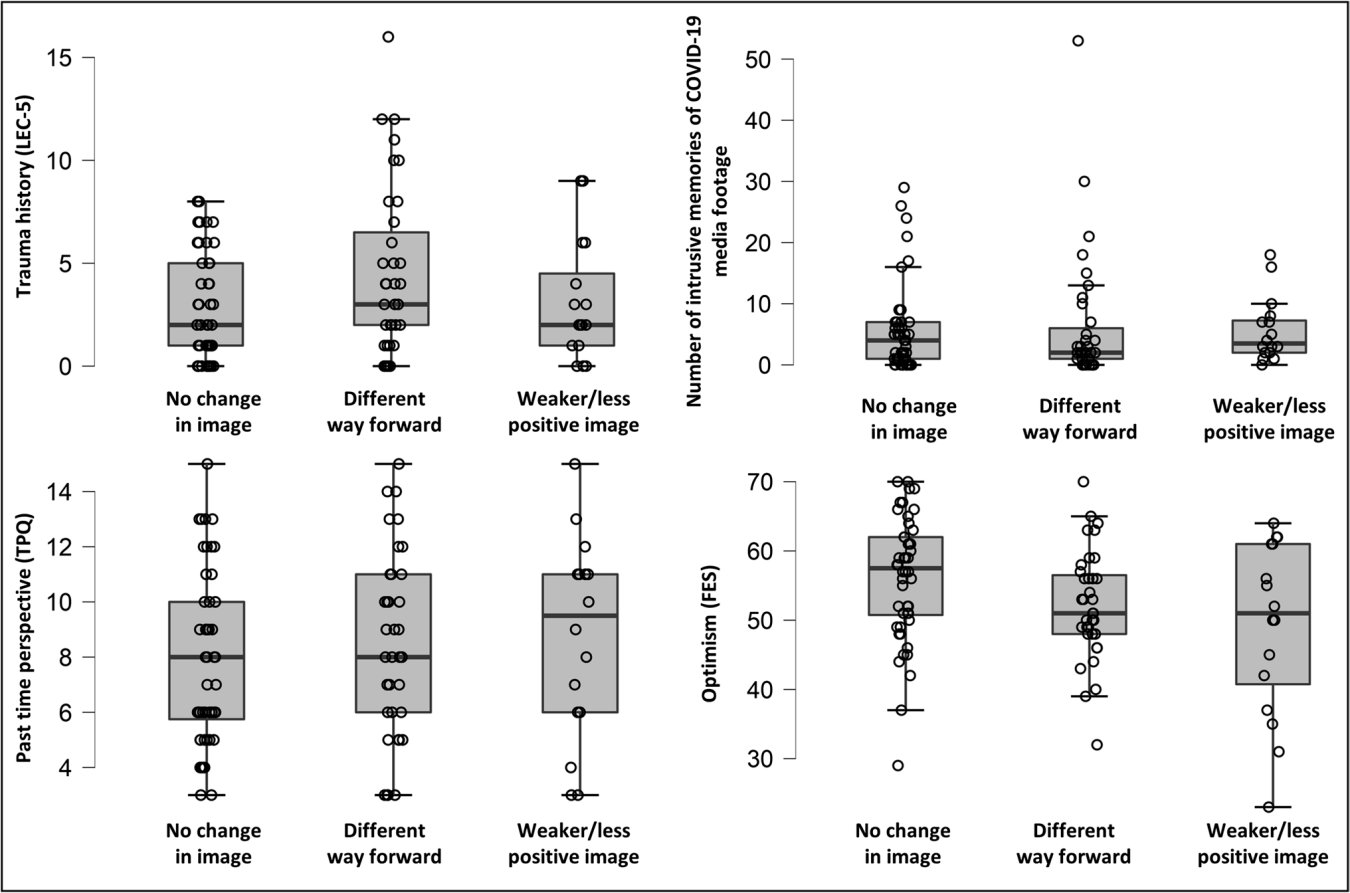
Change of mental image content (researcher-rated) and trauma history (LEC-5), number of intrusive memories of COVID-19 media footage, past time perspective (TPQ), and optimism (FES) in study 2

When comparing participants whose future image content had not changed, changed to a different/adaptive way forward, or became weaker or less positive, no differences emerged for the number of prior traumas (*H*(2) = 3.785, *p* = .151), number of intrusive memories of COVID-19 media footage (*H*(2) = 1.221, *p* = .543), or ratings of past time perspective (*H*(2) = 0.944, *p* = .624). However, there was a significant difference in optimism between categories (*F*(2) = 4.013, *p* = .021, η²_p_ = .077). Specifically, participants whose future image content had not changed during the pandemic reported significantly higher optimism than participants whose future image content was weaker/less positive during compared to the pandemic (*p* = .028, *d* = 0.704), see Fig. 4 for details.

## Study 2 discussion

Similar to Study 1, the content of future self-images (researcher-rated) did not change for the majority of participants (c. 50%), although for some (c. 35%) they changed to a different/adaptive way forward, and for the remaining participants (c. 15%) the images became weaker/less positive. Again, similar to Study 1, participants rated their future self-images as less positive and more vivid and with fewer other people present during (compared to before) the COVID-19 pandemic. Additionally, they indicated that they saw themselves as older (compared to their current age) in their future self-images during compared to before the pandemic. We did not replicate the findings of Study 1 regarding significant associations between the change in valence of future images and other trauma-related variables (i.e., prior trauma, number of intrusive memories of COVID-19 media footage, past time perspective). However, in Study 2 we replicated the finding that the more optimistic participants were, the less negative their future images became. The same pattern emerged for researcher-rated categories regarding changes in participants’ future image content. Specifically, whereas there were no differences between categories for prior trauma, the number of intrusive memories of COVID-19 media footage or past time perspective, participants whose future images changed to a weaker/less positive version were less optimistic than participants whose future images had not changed from before to during the pandemic.

## General discussion

Our first aim was to explore whether the content and characteristics of future self-imagery in young people shifted from before to during the pandemic. Across two studies with independent samples, participants rated their future self-images as less positive *during* relative to *before* the pandemic. This pattern was observed irrespective of the stage in the pandemic in Sweden; i.e., during both the second/third wave (October 2020-March 2021, Study 1) and the fourth wave (October-December 2021, Study 2) – highlighting a consistent impact of the pandemic on the way in which young people envisaged their future.

Our second aim was to determine whether changes in future self-imagery were linked to other (trauma-related) variables. In both stages of the pandemic, reduced positivity of future self-imagery was smaller in individuals who were more optimistic. Interestingly, reduced positivity was also associated with other trauma-related variables (e.g., prior trauma, number of intrusive memories of COVID-19 media footage, past time perspective) in the sample recruited earlier in the pandemic (i.e., second/third waves), but not in the fourth wave. It is possible that the very experience of the pandemic changed in innumerable ways over the course of time (e.g., owing to fluctuations in the level of restrictions, level of threat posed by the virus, case numbers, number of people dying from COVID-19, etc.) which may account for differences in the relationships observed at different timepoints. It is also possible that the associations with other trauma-related variables found in Study 1 (which was exploratory and intended for generating hypotheses) were not robust. Further research is needed to test if the findings are replicated in different samples.

Our studies provide some important initial clues regarding ways in which adverse events may be linked to changes in young peoples’ future self-imagery. Although in both samples most participants’ future images did not change in content, or changed to a different/adaptive (in terms of restricted possibilities during the pandemic) way forward, future self-images became weaker (e.g., less vivid, less likely) or less positive for a considerable minority of participants (i.e., c. 30% in Study 1 and c. 15% in Study 2, changes in content were rated by the researcher). Interestingly, the majority of participants rated their future images as less positive during compared to before the pandemic, indicating that even if image content remained similar, people may experience less anticipatory pleasure when imagining them during the pandemic. While future self-images during (compared to before) the pandemic were rated as less positive, they were also rated as more vivid (in both studies). A combination of less positive but more vivid future imagery could potentially lead to more severe consequences for psychological well-being.

A key finding is that adverse consequences of the pandemic for future self-imagery were lower for individuals with higher optimism, extending previous research on the potential benefits of optimism (Dricu et al., 2020; Singh et al. 2020; Kress & Aue, 2017) in various areas including mental and physical health. We note that our studies did not investigate causal relationships, leaving it unknown whether less optimistic individuals engaged in less positive mental imagery during the pandemic, or whether a lack of positive mental imagery during the pandemic may have impaired optimism (Blackwell et al., 2013; Ji et al., 2017). Furthermore, this finding is in line with earlier research in Germany and the US showing an association between how individuals *think* about the future during the COVID-19 pandemic and psychological well-being (Niziurski & Schaper, 2021), a construct closely related to optimism. Future research is needed to investigate the role of mental *imagery* (specifically positive future self-imagery) in this relationship.

A key limitation of both studies is that participants’ ratings of the characteristics (e.g., valence) of their imagery prior to the pandemic were made retrospectively. Accordingly, we cannot rule out the possibility of recall bias, nor the possibility that ratings were influenced by participants’ current emotional state. In addition, our retrospective design could also account for the lower vividness ratings of future imagery before the pandemic; that is, pre-pandemic imagined events might have been more abstract by virtue of the fact they relate to a future that is less realistic than the one that will follow from the pandemic. Such shortcomings would be eliminated by the use of a longitudinal design in future work. That said, it is difficult to imagine how this could be achieved – that is, how researchers could anticipate an upcoming pandemic. Nonetheless, we acknowledge the reliance on retrospective ratings as a limitation. In addition, we note that some of the coding schemes employed were developed for the purpose of the current project. Accordingly, kappa values varied considerably, which may render some of the coding results less reliable than they would have been had an established coding scheme been available. In addition, we note that we did not pre-register our analyses for Study 1, which was intended to generate hypotheses for Study 2, and thus recommend that the results of Study 1 be interpreted with caution.

These limitations are offset by notable strengths. First, to our knowledge this study is one of the first to investigate how future imagery is affected by a significant and ongoing stressor – in contrast to studies investigating future thinking/imagery in PTSD samples, who may have experienced a trauma many years earlier. Second, we have extended the literature and built on studies examining future imagery in the context of trauma and stressors (e.g., Kleim et al., 2014) to specifically investigate future *self*-imagery. Finally, whilst Study 1 was exploratory, our (pre-registered) replication of the results obtained in Study 1 in an independent dataset (i.e., Study 2) represents a key methodological strength.

Whilst we employed an unselected sample, the findings nonetheless have clinical implications. By suggesting that future self-imagery is influenced by stressful events (and the ongoing pandemic specifically), the findings raise the possibility that brief, simple clinical interventions that aim to boost positive imagery, particularly for individuals who are less optimistic about the future or engage less in positive future self-imagery, may have utility. Indeed, evidence is emerging in support of the effectiveness of novel interventions targeting mental imagery in depressed adolescents (e.g., Pile et al., 2021). Along similar lines, a recent study found that a brief (4 session) intervention incorporating mental imagery delivered by telephone reduced depression in isolated older adults during the COVID-19 pandemic (Pellas et al., 2022a, b). The possibility that brief, easily administered, scalable interventions targeting mental imagery could effectively bolster positive future imagery in the context of the ongoing pandemic is an exciting clinical possibility that awaits empirical test.

In sum, in two studies conducted at different stages of the pandemic, young people rated their future self-images as less positive *during* relative to *before* the pandemic. Optimism played a buffering role: for individuals who were more optimistic, imagery valence ratings became less negative over time. Whilst preliminary, the findings suggest that brief, novel interventions aiming to boost positive future imagery may hold promise to enhance the mental health of young people in the context of the COVID-19 pandemic.

### Supplementary information

Below is the link to the electronic supplementary material.

ESM 1 (DOCX 160 KB)

## Data Availability

The datasets generated during and/or analysed during the current study are available from the corresponding author on reasonable request.
