# Supplementary material for: Future self-imagery of young people in Sweden during the COVID-19 pandemic: an exploratory mixed methods analysis
Source: Curr Psychol. 2022 Dec 20;43(16):15020–34. doi: 10.1007/s12144-022-04100-z (PMC9765368; doi:10.1007/s12144-022-04100-z)
Supplement: Supplementary file 1 — (DOCX 160 KB) [file 12144_2022_4100_MOESM1_ESM.docx]

**Supplementary Information**

**Future self-imagery of young people in Sweden during the COVID-19 pandemic:**

**An exploratory mixed methods analysis**

**_________________­­­_________________________________________________**

**Supplementary Methods**


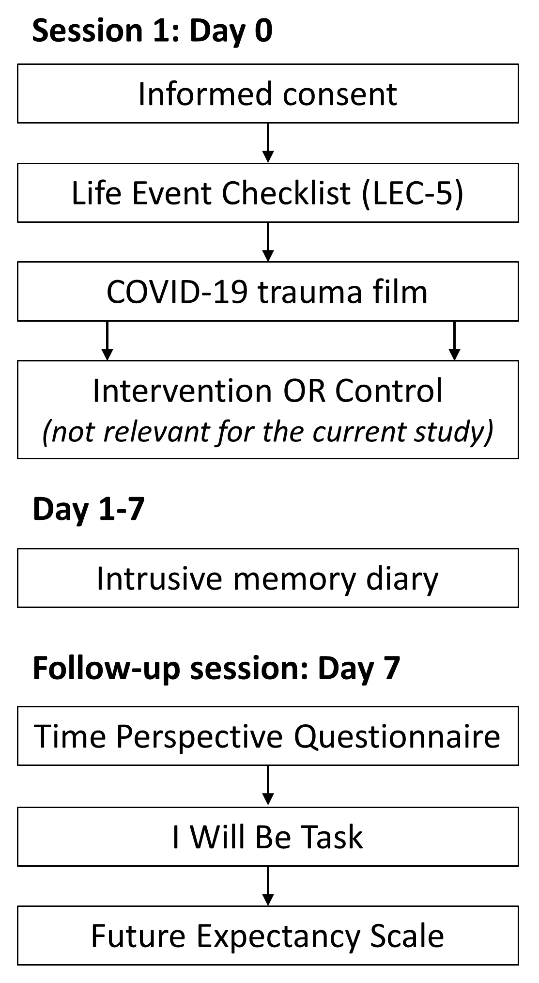


**Supplementary Figure S1.** Flow chart illustrating study procedures.

**Supplementary Table S1.** Coding scheme for ‘I Will Be’ (future identity) statements, developed by Rathbone et al., 2016.

| Category | Explanation & Examples |
| --- | --- |
| 1 Acquiring property | Home-owner etc. |
| 2 Activities | Gardener, traveller, charity volunteer (activities that do not require ‘learing’– these would be classed as skill development) |
| 3 Aging | Relating to getting older |
| 4 Education-university | Relating to university, degrees, graduating |
| 5 Fall in love | In love, fall in love |
| 6 Family | Family is used for being a daughter or a son, aunt or uncle, or about family in general |
| 7 Finance self-improvement | Relating to being wealthy, rich, well-paid, anything associated with money |
| 8 Friendship | Relating to being friends |
| 9 Grandchildren | Grandmother, grandfather, grandparent |
| 10 Happy | Has own category (even though fits into trait – self-improvement – should be coded separately) |
| 11 Health | Has to do with fitness, illness (mental and physical), health in general |
| 12 Busy | Has own category as P2 occurrences |
| 13 Possessions | Anything to owned that isn’t property (which is coded as ‘acquiring property’) or job-related (e.g. ‘the owner of a cafe’ would be coded as job-specific) |
| 14 Move | Relating to moving to house or country |
| 15 Job-general | If job is discussed in broad terms (e.g. do a job I enjoy, be good at my job) |
| 16 Job-specific | If specific details about job/occupation are given (e.g. be a psychologist, writer, doctor) |
| 17 Learn languages | Although skill development this is a separate category to allow cross-cultural comparisons |
| 18 Marriage | Partner, husband, wife, married |
| 19 Military | Relates to military service |
| 20 Other | Response does not fit into any other category |
| 21 Parenthood | Father, mother, parent |
| 22 Physical appearance | Relating to looks, weight, attractiveness |
| 23 Relationship | Relationship used for boyfriend or girlfriend, if not explicitly about marriage (partner is classed as marriage) |
| 24 Religion | Any mention of religion |
| 25 Self-improvement | Abstract statements relating to being better in the future (e.g. clever, a better person, academic) |
| 26 Skill development | Skill development if the future self involves learning (e.g. driving a car, cooking, dancing), but ‘activity’ if not (e.g. giving to charity, going travelling) |
| 27 Successful | Has own category (even though fits into trait – self-improvement – should be coded separately) |
| 28 Trait-general | If not associated with self-improvement (e.g. negative or neutral traits, such as lazy, stressed, different) |

**Supplementary Table S2.** Coding scheme for changes in the content of images related to participants’ future self identity.

| Category and code | Description |
| --- | --- |
| No change (1) | The image content during the pandemic is the same as before the pandemic  *Examples*: Participant gives the same image description for both time points, ‘Exactly the same’ |
| Different/adaptive way forward (2) | The image content has changed to a different/adaptive way forward (the image has been adjusted to one that is possible under pandemic conditions but is similar in valence/quality)  *Examples*: the image includes fewer people, has been adjusted to e.g. a digital version |
| Weaker/less positive image content (3) | The image content is weaker/less positive during compared to before the pandemic  *Examples*: the image is described as less clear, less likely to happen, to happen further away in the future or the image is less positive |

**Supplementary Table S3.** Coding scheme for specificity of images content related to participants’ future identities (developed by LS and CR for the current study)

| **Category (code)** | **Description** |
| --- | --- |
| Specific image  (1 = yes, 0 = no) | Is a specific image described?  *Examples of images*: the coder can imagine a specific image based on the description (‘I see myself as a doctor at a health care center’)  *Examples of no image*: traits (‘I will be healthier’), general descriptions of extended events (‘I want to be happy, enjoy my life’) |
| Who  (1 = yes, 0 = no) | Does the image description include who is in the image?  *Examples*: I see myself, a doctor, a group of friends |
| Where  (1 = yes, 0 = no) | Does the image description include where the image takes place?  *Examples*: at a work place, in Mora, on the sofa |
| When  (1 = yes, 0 = no) | Does the image description include when the image takes place?  *Examples*: When my knee is better, every morning |
| What  (1 = yes, 0 = no) | Does the image description include what is happening in the image?  *Examples*: I work outside on the field, intubating a patient in the ED |

**Supplementary Table S4.** Cohen’s Kappa for all parts included in the different qualitative coding schemes

| **Coding scheme (category)** | **Cohen’s Kappa** |
| --- | --- |
| *Study 1* | |
| I Will Be statements (related to social situations) | .839 |
| I Will Be statements (related to occupational situations) | .853 |
| I Will Be statements (related to other important situations) | .658 |
| Image content changes | .693 |
| Specificity (specific image) | .277 |
| Specificity (who) | .619 |
| Specificity (where) | .854 |
| Specificity (when) | .546 |
| Specificity (what) | .190 |
| *Study 2* | |
| Image content changes | .450 |

**Supplementary Results**

**Examples of young people’s future self-identities before the COVID-19 pandemic**

The most common future self-identity statements were related to: ‘Parenthood’ and ‘Marriage’ for social identity; ‘Job - specific’, ‘Job - general’, and ‘Education – university’ for occupational identity; and ‘Health’, ‘Self-improvement’ and ‘Activities’ for other important identity (see further details and examples in Table S5 below).

**Supplementary Table S5.** Content of future self-identities per category (social, occupational, other important identity) before the COVID-19 pandemic in study 1.

|  |  | |
| --- | --- | --- |
| **Category** | ***Count (n)*** | **%** |
| *Social future identity* |  |  |
| Parenthood | 25 | 33.8 |
| Marriage | 11 | 14.9 |
| Friendship | 9 | 12.2 |
| Relationship | 8 | 10.8 |
| Self-improvement | 4 | 5.4 |
| Family | 3 | 4.1 |
| Fall in love | 2 | 2.7 |
| Grandchildren | 2 | 2.7 |
| Move | 2 | 2.7 |
| Other | 2 | 2.7 |
| Activities | 1 | 1.4 |
| Aging | 1 | 1.4 |
| Education – university | 1 | 1.4 |
| Skill development | 1 | 1.4 |
| Successful | 1 | 1.4 |
| Trait – general | 1 | 1.4 |
| *Occupational future identity* |  |  |
| Job – specific | 41 | 55.4 |
| Job – general | 14 | 18.9 |
| Education – university | 13 | 17.6 |
| Self-improvement | 2 | 2.7 |
| Successful | 2 | 2.7 |
| Other | 1 | 1.4 |
| Skill development | 1 | 1.4 |
| *Other important future identity* |  |  |
| Health | 16 | 21.6 |
| Self-improvement | 15 | 20.3 |
| Activities | 14 | 18.9 |
| Happy | 6 | 8.1 |
| Skill development | 5 | 6.8 |
| Move | 3 | 4.1 |
| Other | 3 | 4.1 |
| Friendship | 2 | 2.7 |
| Job-specific | 2 | 2.7 |
| Religion | 2 | 2.7 |
| Acquiring property | 1 | 1.4 |
| Aging | 1 | 1.4 |
| Possessions | 1 | 1.4 |
| Learn languages | 1 | 1.4 |
| Relationship | 1 | 1.4 |
| Trait – general | 1 | 1.4 |

**References**

Rathbone, C.J., Salgado, S., Akan, M., Jelena, H., & Berntsen, D. (2016). Imagining the future: A cross-cultural perspective on possible selves. *Consciousness and Cognition*, *42*, 113–124. https://doi.org/10.1016/j.concog.2016.03.008
